# Searching for G: A New Evaluation of SPM-LS Dimensionality

**DOI:** 10.3390/jintelligence7030014

**Published:** 2019-06-28

**Authors:** Eduardo Garcia-Garzon, Francisco J. Abad, Luis E. Garrido

**Affiliations:** 1 Facultad de Psicología, Universidad Autónoma de Madrid, 28049 Madrid, Spain; 2 Facultad de Psicología, Pontificia Universidad Católica Madre y Maestra, Santo Domingo 10109, Dominican Republic

**Keywords:** Raven matrices, Standard Progressive Matrices test, dimensionality, bi-factor, parallel analysis, target rotation, exploratory graph analysis

## Abstract

There has been increased interest in assessing the quality and usefulness of short versions of the Raven’s Progressive Matrices. A recent proposal, composed of the last twelve matrices of the Standard Progressive Matrices (SPM-LS), has been depicted as a valid measure of *g*. Nonetheless, the results provided in the initial validation questioned the assumption of essential unidimensionality for SPM-LS scores. We tested this hypothesis through two different statistical techniques. Firstly, we applied exploratory graph analysis to assess SPM-LS dimensionality. Secondly, exploratory bi-factor modelling was employed to understand the extent that potential specific factors represent significant sources of variance after a general factor has been considered. Results evidenced that if modelled appropriately, SPM-LS scores are essentially unidimensional, and that constitute a reliable measure of *g*. However, an additional specific factor was systematically identified for the last six items of the test. The implications of such findings for future work on the SPM-LS are discussed.

## 1. Introduction

The Standard Progressive Matrices (i.e., SPM [1]), in any of its forms, constitutes one of the most applied tests for measuring general intelligence (*g*). Due to its considerable length (60 items), there has been a growing interest in developing short versions of this test. Unfortunately, the available short versions—such as the Advanced Progressive Matrices tests (i.e., APM)—present substantial shortcomings [2]. Consequently, [2] proposed the SPM-LS, a new short version of the SPM test based on its last, most-difficult 12 matrices of this test. These items consist of non-verbal stimuli where each item presents a single correct answer and seven distractors. In its recent validation, the SPM-LS scores were analysed using exploratory and confirmatory factor analyses as well as item response theory models as follows: After concluding that the SPM-LS scores were sufficiently unidimensional, individual responses were modelled with the 1 to 4 parameter logistic models. Additionally, a three-parameter nested logistic model was applied to recover relevant information from responses to the different distractors. Remarkably, the original authors concluded that the SPM-LS was a superior alternative to the APM test ([2]; p.113), and encouraged other researchers to re-analyse this dataset by making it publicly available and by opening a call for papers on the matter in the Journal of Intelligence. 

As part of this call, this investigation will re-evaluate [2] claim of SPM-LS being essentially unidimensional. This claim is vital to understand if SPM-LS represents a valid measure of *g* and represent a necessary assumption for many of the following analysis presented by the original authors. As [2] acknowledged that “SPM-LS may not be a purely unidimensional measure” (p.114), we decided to analyse SPM-LS dimensionality by expanding the original approaches with the application of network-based exploratory analysis and bi-factor modelling.

### 1.1. On the Progressive Matrices Dimensionality

**Figure 1:**
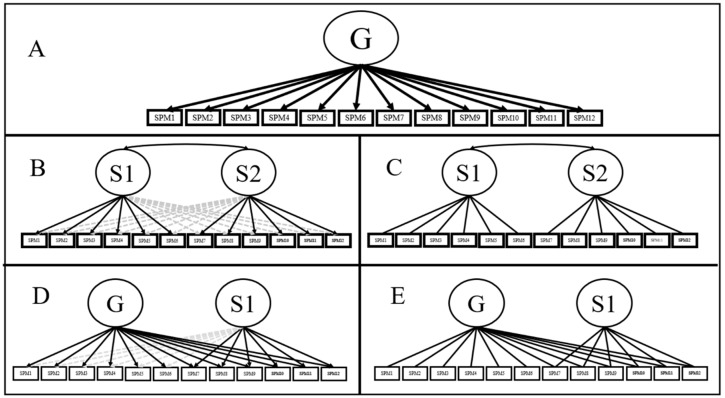
Schematic representation of theoretical SPM-LS models: (**A**): Unidimensional model; (**B**) Exploratory bi-dimensional model; (**C**): Confirmatory bi-dimensional model; (**D**): Exploratory bi-factor model; (**E**): Confirmatory bi-factor model. Arrows in black represent estimated paths for CFA models, and untargeted loadings in EFA models. Grey arrows represent targeted (minimised) loadings during EFA target rotation.

Few consensuses are more extended in the intelligence literature than the belief that the SPM test [1] represents a consistent measure of general intelligence (*g*; Panel A, Figure 1). Even though this claim has received overwhelming support in the literature [3,4,5], other authors have considered general intelligence to be a broader construct to be measured with different tasks and item formats [6]. Be that as it may, support for strict unidimensionality has historically been equivocal for short SMP versions such as the APM test. As early as 1981, some authors found evidence of an orthogonal two-factor model [7,8] were among the first authors to suggest that a nuisance factor, corresponding to a “speed factor”, could be found for APM scores (Panel C, Figure 1). [3] found that the two-factor proposed in [2] fitted the data better than the single factor model if the inter-factor correlation was estimated. Nevertheless, the high magnitude of this correlation (i.e., 0.89; Panel B, Figure 1; [3]), in conjunction with the inspection of fit statistics, was taken as evidence in favour of a unidimensional model. Since then, other authors on the field have supported [3] conclusions [4,5].

Recent applications of bi-factor modelling offered new insights regarding the dimensionality of the APM, as well as the role of potential secondary factors (Panel E, Figure 1). As the bi-factor model simultaneously estimates a general plus several orthogonal specific factors [9], it provides a clear separation of such different sources of variation. Noteworthy, as specific factors only account for a variance that is residual to the general factor [10], the bi-factor model can shed light about APM scores being affected by other sources of variation in addition to *g*. Indeed, APM scores do not represent a perfect measure of *g* and that alternative tests (such as Arithmetic Applications from the Weschler Adult Intelligence Scale included in the Minnesota Study of Twins Reared Apart [11]) were more strongly loaded by *g* in some specific datasets [12]. Moreover, approximately 50% of the APM true variance could be related to *g*, with 10% belonging to specific factors, and as much as 25% related to test specific variance [12]. Confirmatory bi-factor models (i.e., BCFA) also presented a better fit to the data than the unidimensional model in alternative applications such as the Coloured Progressive Matrices test (an adaptation of the APM test to children from five to 11 years old; [13]).

Most recently, the presence of additional dimensions accounting for speed factors (as well as other effects such as item position) in APM scores [14] has been linked to specific learning types [15] as well as developmental differences [16]. In either case, such evidence reflects these factors possibly being of theoretical interest. Nevertheless, the presence and nature of these additional factors in APM scores is still a matter of contention.

### 1.2. Modern Approaches Towards Dimensionality Assessment

Most authors have generally based their decisions regarding the unidimensionality of the SPM scores either by applying eigenvalue-based dimensionality assessment methods (i.e., parallel analysis), by comparing fit statistics from CFA models (i.e., comparing the Comparative Fit Index) or by inspecting general factor reliability (i.e., Cronbach’s α). Unfortunately, these three strategies have substantial shortcomings: Firstly, parallel analysis could hide relevant sources of variation while overestimating the presence of a single factor [17]. Also, its estimation is substantially affected by the response patterns when analysing tetrachoric and polychoric correlation matrices under limited sample size [18]. Secondly, CFA models could hide severe misspecification issues and result in biased parameter estimation [19,20]. Accordingly, CFA model-based reliability estimations could also be highly biased [21]. Thus, exploratory structures should be preferred in many cases [18,19]. We aim to resolve these issues by complementing these analyses with a new technique for dimensionality assessment (EGA) and the novel investigation of different exploratory factor models for the SPM-LS test.

#### 1.2.1. Parallel Analysis

Parallel analysis is one of the main tools for dimensionality assessment [17,22,23]. Either when based on principal component or factor analysis solutions, parallel analysis has repeatedly been shown to optimally detect the true underlying unidimensionality in simulation studies [23,24,25]. However, parallel analysis is also fallible [18,23], with different conditions affecting each version of this procedure [17,22]. Principal component factor analysis is more reliable than the factor analysis alternative for structures with a small number of factors and binary data [17,22]. Unfortunately, it tends to wrongly suggest a single component to be retained if high factor correlations are present (as expected to occur in SPM-LS; [3]). On the other hand, factor analysis-based parallel analysis could be misleading if factors are not well defined (i.e., factor loadings < 0.40; [17]), which is indeed a plausible scenario for SPM-LS scores based on [12] depiction of APM variance partition. Additionally, either method presents difficulties in recovering the true dimensionality if samples < 500 are analysed (the size of [2] dataset; [17,26]). Finally, binary and categorical items presenting highly unbalanced categories (e.g., where the correct response represents 80–90% of the observed responses) could strongly affect parallel analysis performance [18,27,28].

#### 1.2.2. Exploratory Graph Analysis

Exploratory Graph Analysis (EGA) is a statistical procedure that assesses latent dimensionality by exploring the unique relationships across pairs of variables (rather than the inter-item shared variance, as in common factor analysis; [29]). To do so, a sparse Gaussian Graphical Model is estimated (i.e., GGM) over the K precision matrix. K is the inverse of the inter-item variance-covariance matrix (i.e., $K=\Sigma^{-1}$; [30]) and it contains the partial correlations across pairs of observed variables. The sparse GMM is estimated by applying a penalization function (a common method is to select the GMM which minimises the extended Bayesian Information Criterion). After the GLASSO GMM is estimated, a walktrap clustering algorithm is applied to detect the optimal number of clusters in the network and to assign each item to a single dimension [21]. This algorithm, namely the combination of GLASSO GMM and walktrap clustering, has received the name of EGA. Although alternative versions of EGA exist, such as EGA with the triangulated maximally filtered graph approach (EGAtmfg), the former is preferred when high correlations between factors are expected (being the case for SPM-LS) [21].

EGA has been successfully applied to investigating the dimensionality of constructs such as personality [31], intelligence [32], and demonstrated to be as effective as parallel analysis when recovering true dimensionality under dichotomous data [17]. Nonetheless, EGA should be able to detect the number of underlying dimensions equal to or better than parallel analysis, even under suboptimal conditions (limited sample size; [17]). EGA is not presented as a substitute for techniques such as parallel analysis, but rather as a complementary tool to be studied in combination with them [17]. Accordingly, if parallel analysis results in indications of multidimensionality, researchers could benefit from exploring new techniques based on network analyses [30].

#### 1.2.3. Exploratory Bi-factor Modelling

A review of the SPM literature has shown that two main factors models have been of interest: a unidimensional [2,4] and a multidimensional (bi-dimensional) solution [8]. Thus, it is legitimate to question to what extent specific sources of variance detected by parallel analysis or EGA could provide additional, meaningful information beyond *g*. In this sense, the bi-factor model should be the model to be evaluated [32,33]. The bi-factor model has been depicted as the best-suited model for assessing variance partition, to examine whether a structure is sufficiently unidimensional, and to measure the incremental value of potential specific factors [21,32,33]. When assessing estimated general factor strength, factor reliability should be compared using the omega hierarchical statistic ($\omega_{H}$) [21,32]. Additionally, and to test the hypothesis of sufficient unidimensionality, the Explained Common Variance (i.e., ECV) and the Percentage of Uncontaminated Variances (PUC) should be compared altogether with $\omega_{H}$ for confirmatory models [34,35]^1^.

All model-based statistics are computed from a standardised factor analysis solution [32,36]. Therefore, it is necessary to ensure a proper estimation of the underlying bi-factor model in order to obtain unbiased reliability and ECV estimates. Given the difficulties for CFA models to recover complex structures (such as the bi-factor model) under realistic conditions (when cross-loadings are expected to occur; [19]), the bi-factor CFA models are often expected to produce biased parameter estimation [33]. In this context, exploratory alternatives such as EFA or Exploratory Structural Equation Modeling (i.e., ESEM) are becoming more and more widespread [37,38]. As these techniques offer model fit assessment while not imposing restrictions on the factor pattern matrix, they provide the modelling advantages of CFA while improving parameter estimation [18,39].

Exploratory bi-factor analysis (BEFA; Panel D, Figure 1) is a widely applied, compelling alternative to confirmatory bi-factor models [40]. The unique distinction between a BCFA and BEFA is that the latter allows the presence of cross-loadings for all specific factors [36] while maintaining the remaining characteristics (i.e., orthogonality between all factors). As each specific factor is still expected to be loaded by at least three indicators, variance partition, as well as the remaining BCFA characteristics, are present in a BEFA model [35]. However, how to approximate BEFA models is still a matter of debate. One of the most promising alternatives is via bi-factor target rotation, a technique applied in the BIFAD [10], the PEBI [41], or the SL-based iterative target rotation (SLi and SLiD algorithms; [36,38]).

In bi-factor target rotation, factor loadings to be minimised in the rotation procedure (i.e., items expected to have near-zero magnitude in the rotated loading matrix) are identified by giving them a zero value in the target matrix. As a convention, as general factor loadings are always freed (as each loading is expected to have a substantial load on this factor). The main issue then is to identify which loadings should be freed in the target rotation for the specific loadings. Conveniently, empirical cut-off points such as promin [42] or the procedure applied in SLiD algorithm [36] are able to select which loadings to be fixed based on each factor ’s loadings distribution, and to prevent researchers from deciding on applying inappropriate fixed cut-off points (such as fixing all λ<0.20; [36]). As an example, SLiD has been demonstrated to accurately recover bi-factor models in conditions under realistic conditions (i.e., cross-loadings or specific loadings of near-zero value), and to outperform more well-known methods such as the Schmid-Leiman orthogonalization, and the family of analytic rotations [43,44]. Promin-based algorithms (i.e., PEBI) has also been depicted as a compelling alternative and an improvement over alternative algorithms such as BIFAD [42]. Additionally, as the use of empirically defined target rotation is expected to improve parameter estimation, the estimation of general omega hierarchical, ECV and other model-based reliability estimates is also anticipated to be improved.

### 1.3. SPM-LS Dimensionality

SPM-LS dimensionality was evaluated by using a combination of parallel analysis, EFA and CFA results [2]. However, due to the limited sample size and the unbalanced responses patterns, parallel analysis results presented by the authors should be examined with caution. As the authors acknowledged, SPM-LS data presented some strong ceiling effects, when “10.4% of the sample had a perfect score of 12” [2] (p.114). This situation could have resulted in suboptimal performance of parallel analysis. In the results section, the authors declared that up to five factors should be retained via factor analysis parallel analysis. Additionally, and due to the large ratio of the first to second eigenvalue (5.92 to 0.97), evidence of a robust general factor was said to be found [2]. However, as factor analysis parallel analysis could be more unreliable than its principal-component alternative for the study at hand (due to limited sample size and the binary nature of the data), the results of both techniques should have been taken into consideration (e.g., when computing ratios of eigenvalues). 

The authors additionally reported that no evidence of relevant specific factors was identified, as factor pattern loadings on unreported solutions including two to five factors were not in line with any theoretical expectation (i.e., “were uninterpretable”; [2], p. 112). However, the authors did not report the structures tested, or if models combining general and specific sources of variation (i.e., bi-factor) were estimated. Lastly, as global fit indexes suggested an adequate fit for the unidimensional model (i.e., even though RMSEA was as high as 0.079) and the general factor was considered as reliable ($\omega_{H}=0.86$), the authors concluded that the SPM-LS scores could be considered essentially unidimensional [2] (p.112). In this investigation, this claim will be revisited by a more nuanced inspection of SPM-LS scores by applying traditional methods (exploratory and confirmatory unidimensional and bi-dimensional factor models) as well as two recently developed methods for assessing and validating multidimensional scales (EGA and bi-factor exploratory modelling).

## 2. Materials and Methods 

### 2.1. Instrument and Data

The SPM-LS scores are those made publicly available by [2] for this special edition. In detail, the sample is composed of the answers of 499 undergraduate students who responded to the SPM-LS. The SPM-LS consists of the last 12 matrices the Standard Progressive Matrices [1] (i.e., those of greatest difficulty). Noteworthy, even though these items could be considered as polytomous, and essential information could be retrieved if they were treated as such [2], it is common to score them as dichotomous items: either a respondent identified the correct answer or not according to the item key provided by the authors. Accordingly, the tetrachoric correlation matrix was here studied. In this application, respondents had no time limit to complete the 12 items and were encouraged to respond to each item. Accordingly, no missing data were observed.

### 2.2. Statistical Analysis Plan

The following analysis will be performed to inspect the factor structure of the SPM-LS: Firstly, the dimensionality of the SPM-LS will be assessed applying both, principal component and factor analysis parallel analysis. Secondly, these results will be contrasted with those of EGA. If the SPM-LS is regarded as multidimensional, the hypothesis of essential unidimensionality will be tested by inspecting a series of unidimensional, exploratory and confirmatory bi-dimensional and bi-factor models (Figure 1). These models would be compared in terms of model fit, factor pattern results, $\omega_{H}$ and ECV, and PUC values (when possible). To estimate BEFA models, a bi-factor target rotation would be defined from bi-dimensional EFA solution, using the empirical cut-off point definition algorithm included in SLiD [36] and the promin cut-off estimation [42].

Most analyses were conducted in R 3.5.2. [45] in a reproducible manner using the rmarkdown [46] and the papaja [47] packages. The correlation matrix was obtained using the *cor_auto ()* function in the qgraph package [48], which provided similar results to the *tetrachoric ()* function from the psych package [49]. Principal component and factor analysis were conducted using the *fa.parallel ()* function in the psych package [49]. EGA was applied using the EGA package [50]. EFA and CFA models were computed using the lavaan package [51]. Cronbach’s α and omega estimates were computed from the *reliability ()* function from the semTools package [52] following current recommendations on the field [53]. EFA models were rotated using oblique target rotation using the gradient projection algorithm included in the GPArotation package [54]. Bi-factor target was defined using the promin rotation [42] and the algorithm included in the SLiD [36]. The bi-dimensional EFA model was computed using minimum residual as the extraction method and target rotation towards the expected EGA solution. ESEM models for estimating bi-dimensional EFA and bi-factor EFA models with a free residual correlation were fitted in Mplus 7.3. Scripts for reproducing all analyses (i.e., main text, Appendix A and Appendix B results) can be found as Supplementary Data.

## 3. Results

### 3.1. Descriptive Analysis

**Figure 2:**
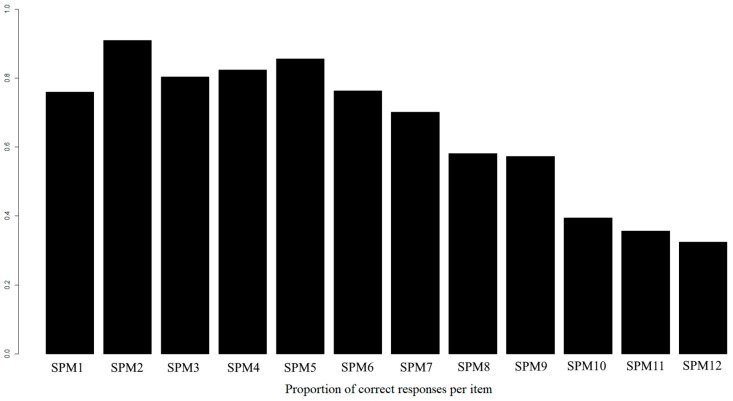
Proportion of correct responses as a function of item location in the SPM-LS.

A characteristic of the SPM-LS is that the chosen items represent the most difficult items from the SPM. However, the proportion of correct responses did not monotonically decrease as a function of item position (Figure 2), as it could be somewhat expected. The first six items (SMP1 to SMP6) had high correct proportions of correct responses (0.76 < *p_correct_* < 0.91; where *p_correct_* is the observed proportion of correct answers) and were identified to present similar rates of unbalanced response patterns. On the other hand, the last three less than half of the responses collected were correct items (SPM10: *p_correct_* = 0.39; SPM11: *p_correct_* = 0.36 and SPM12: *p_correct_* = 0.32). As said before, these unbalanced response patterns could lead to significant estimation errors in the tetrachoric correlation estimation.

**Figure 3:**
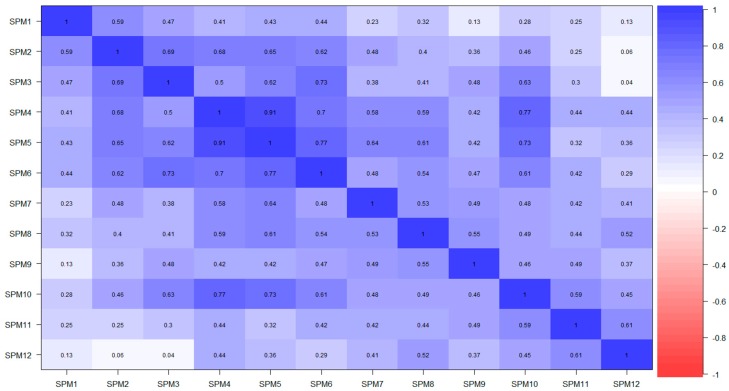
Heatmap of SPM-LS items tetrachoric correlation.

A visual inspection of the tetrachoric correlation matrix (Figure 3) revealed an unusually high correlation between items (*r*_SPM4 – SPM15_ = 0.91), which was substantially larger than the ensuing correlation in terms of magnitude (*r*_SPM5 – SPM16_ = 0.77). In detail, 79.8% of individuals who correctly responded SPM4, also were correct for SPM5. Moreover, 11.8% of respondents who failed SPM4, also failed SPM5. Thus, there was only 8.4% of respondents who failed/gave a correct answer or gave a correct answer/failed SPM4-SPM5, respectively. A visual inspection of the tetrachoric correlation heatmap revealed two distinct blocks of inter-item correlations: The first one between items SMP1 to SPM6, and the second one between items SPM7 to SMP11. Therefore, Figure 3 is indicative of two distinct sources of multidimensionality. Due to the limited sample size, and the highly unbalanced response patterns for items such as SPM2, SPM11, and SPM12, it is noteworthy that the tetrachoric correlations between these items could be affected by significant estimation errors.

### 3.2. Dimensionality Assessment.

**Figure 4:**
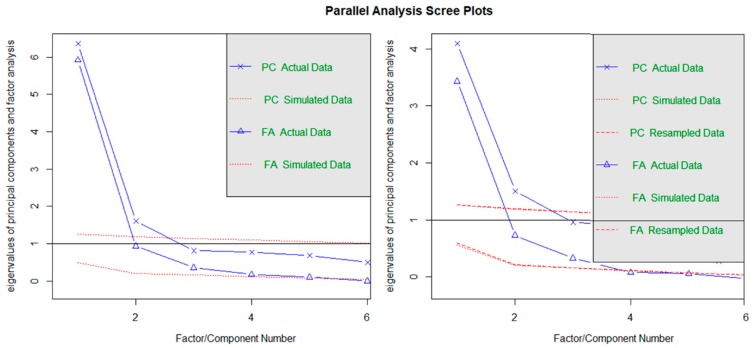
Parallel analysis results: (**a**) Original Principal component and parallel factor analysis with eigenvalue simulated from random normal data; (**b**) Principal component and parallel factor analysis correct eigenvalues obtained from resampling from original data.

We exactly replicated the results provided by [2] when computing parallel analysis over the tetrachoric correlation matrix (using maximum likelihood)^2^ (Left panel, Figure 4; also Figure 1 in [2]). The number of factors to be retained was 5, with eigenvalues of 5.92, 0.93, 0.36, 0.18, and 0.10 (simulated eigenvalues of.52, 0.21. 0.16, 0.12, 0.07). The number of components to be retained was 2, with eigenvalues as of 6.36 and 1.60 (simulated eigenvalues of 1.26 and 1.20). Noteworthy, it was observed that the authors conducted this analysis over the tetrachoric correlation matrix, obtaining the eigenvalues to be compared against those extracted by generating random normal data. However, this strategy is considered highly inadequate [18]. A better strategy when analyzing tetrachoric correlations is to obtain the random eigenvalues by resampling from the observed data. Accordingly, we repeated the analysis with this specification (Right panel, Figure 4). Factor and principal component factor analysis suggested to retain two and three factors/components, respectively: factor analysis parallel analysis showed eigenvalues of 3.43, 0.73 and 0.33 (with resampled eigenvalues of 0.54, 0.20 and 0.15) while principal components PA resulted in eigenvalues of 4.09, 1.51 for the original components (with resampled components of 1.26 and 1.19).

**Figure 5:**
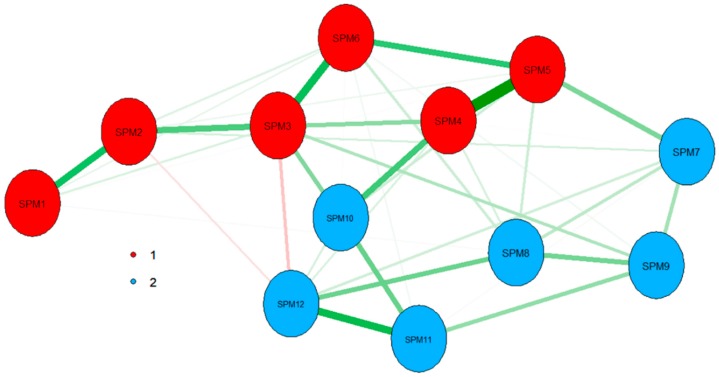
Exploratory Graph Analysis of SPM-LS data. Dimensions and items associated are presented in different colours. Positive partial correlations are depicted in green, with negative partial correlations presented in red. The size of the lines indicates the size of the partial correlations.

Nevertheless, both parallel analysis techniques are suggesting the SPM-LS be multidimensional. The discrepancy between both methods (suggestions of three factors vs two components to be retained) could be due factor analysis-based parallel analysis being more affected by the limited sample size analysed. EGA agreed with principal component parallel analysis and identified two underlying dimensions (Figure 5), one composed of items one to six and the other of items seven to twelve. Moreover, EGA results confirmed that the highest observed partial correlation was observed for the pair SPM4–SPM5. This partial correlation indicates that, after controlling for all the other variables, these items were strongly conditionally dependent. 

Therefore, and after inspecting the tetrachoric correlation matrix and observing the dependence between SPM4–SPM5 items, it was decided to reanalyse SPM-LS dimensionality after aggregating these items. Item parcelling (i.e., aggregating items) have been shown as a valid alternative to deal with residual item covariances [55]. Both techniques of parallel analysis agreed in this re-analysis that two factors should be retained. EGA also resulted in two factors being identified, with a similar distribution than in Figure 5. Therefore, robust evidence from both, parallel analysis and EGA, supported the hypothesis of SPM-LS being bi-dimensional (either when treating the original set of items, or the reduced version combining items SPM4 and SPM5). Analysis details and results of this analysis are presented in Appendix A.

### 3.3. Factor Modelling

The standardised factor solutions for all estimated models are shown in Table 1. Likewise, the fit indices for all estimated models are presented in Table 2. For the sake of comparison, similar models not estimating the residual correlation between SPM4–SPM5 were also computed. Standardised factor loadings and model fit indices of these models without including this residual correlation are presented in Appendix B.

#### 3.3.1. Unidimensional Model

We first replicated the original results with regards to the CFA unidimensional model [2]. We found the same model fit indices (CFI = 0.95, TLI = 0.93, RMSEA = 0.08, SRMS = 0.11). Cronbach’s α = 0.92 and $\omega_{HG}$ = 0.83 also matched those reported. For this model, the high RMSEA and SRMR values suggest questionable fit. Estimating the correlation between SPM4–SPM5 resulted in improved model fit (CFI = 0.95, TLI = 0.94, RMSEA = 0.08, SRMS = 0.11). As expected, the SPM4–SPMP5 correlation was high and positive (ψ=0.69). Accordingly, the remaining presented models will include the estimation of the residual correlation between both items. Additionally, this unidimensional model showed adequate reliability (Cronbach’s α = 0.92; $\omega_{HG}$ = 0.86).

#### 3.3.2. Bi-Dimensional Model

Two bi-dimensional structures were computed. Firstly, an exploratory bi-dimensional model was fitted in order to understand if EFA results supported the idea of a bi-dimensional SPM-LS structure. Secondly, such an EFA structure was tested as a confirmatory model to understand the role of potential cross-loadings present on the data. EFA model fit indexes revealed that this structure provided an excellent fit to the data (CFI = 0.99, TLI = 0.98, RMSEA = 0.04, SRMS = 0.06), improving model fit with respect to the unidimensional case. Additionally, a lower inter-factor correlation of (φ ≈ 0.56) was obtained^3^. The SPM4–SPM5 correlation of this residual correlation (ψ=0.70) was similar to the one observed in the unidimensional model. 

The confirmatory bi-dimensional (CFI = 0.96, TLI = 0.96, RMSEA = 0.06, SRMS = 0.09) presented a better model fit than the unidimensional model, but worse than its exploratory counterpart. Fixing all cross-loadings to zero led to observe a larger factor correlation (φ = 0.82), larger SPM4–SPM5 loadings ($\lambda_{SPM4}=0.89,\lambda_{SPM5}=0.91$), and a diminished residual correlation between them (ψ=0.56). In this case, both factors were considered as reliable if measured by Cronbach’s α standards (factor 1 = 0.91, factor 2 = 0.85), and close to acceptable reliability when inspecting $\omega_{HS}$ (factor 1 = 0.75 factor 2 = 0.70). In conclusion, a bi-dimensional model (either by EFA/CFA based) improved model fit over the unidimensional structure. As indicated by the substantial inter-factor correlation observed in all models, a general factor could play a substantial role in SPM-LS structure. This hypothesis will be explored next via bi-factor modelling.

#### 3.3.3. Bi-Factor Model

Two bi-factor models were tested: a BEFA model fitted using bi-factor target rotation and a BCFA model restricting cross-loadings to zero. Either using the algorithm included in SLiD [36] or a promin-based cut-off [42] resulted in items SPM7 to SPM12 being freed in the specific factor. Noteworthy, as rotation does not affect model fit [29], fit indices for this model were those of the exploratory bi-dimensional structure. The BEFA model (Table 1) presented three main characteristics: (a) The rotation procedure recovered orthogonal factors (even if oblique target rotation was applied), which aligns with the expectations of the bi-factor model; (b) Although the general factor was well-defined (all loadings over $\lambda_{G}>0.30$), SPM11 and SPM12 presented higher loadings on the specific factor ($\lambda_{SSPM11}=0.58,\lambda_{SSPM12}=0.80$) than in the general factor ($\lambda_{gSPM11}=0.44,\lambda_{GSPM12}=0.29$); (c) the residual correlation between SPM4 and SPM5 was similar to the one observed for the unidimensional model (ψ=0.70). With regards to BEFA general factor reliability, it was considered as adequate ($\omega_{HG}$ = 0.80; ECV = 0.74).

The BCFA model showed the best fit indexes from all confirmatory models (Table 2; CFI = 0.98, TLI = 0.97, RMSEA = 0.05, SRMS = 0.07). Both factors were well-defined (all loadings λ>0.30) with SPM4–SPM5 general loadings being stronger than in the BEFA model (as they were inflated due their cross-loadings being fixed to zero). SPM4–SPM5 residual correlation was similar to the one observed in the confirmatory bi-dimensional model (ψ=0.57). Overall, general factor reliability was also adequate ($\omega_{HG}$ = 0.75; ECV = 0.80). Additionally, the associated PUC was (132−42)/132=0.68. Under the presence of PUC<0.80, researchers are recommended that $\omega_{H}>0.70$ and ECV>0.60 be used as benchmarks for considering essential unidimensionality [34]. Therefore, while the BCFA provided an adequate approximation towards SPM-LS multidimensionality, the presence of a strong, reliable general factor also favours that SPM-LS scores be considered as essentially unidimensional. Lastly, the specific factor reliability ($\omega_{HS}$ = 0.31) was in the range of values commonly observed on bi-factor modelling [32,33].

## 4. Discussion

The SPM-LS (Standard Progressive Matrices–Last Series) has been recently proposed as an improved short version of the SPM test [2]. The SPM-LS was treated as an essentially unidimensional measure of *g*, with better psychometric properties than alternative tests such as the Advanced Progressive Matrices test (i.e., APM). On these grounds, [2] proceeded to fit a series of IRT models to study the benefits of studying the nominal responses in the test, acknowledging that mixed results from EFA and CFA results could suggest SPM-LS not being a strictly unidimensional measure. The authors further recommended investigators to conduct additional research on this matter. We aimed to shed light on SPM-LS dimensionality using improving the dimensionality techniques applied (comparing parallel analysis with exploratory graphic analysis results) and by providing a thoughtful exploration of unidimensional, bi-dimensional and bi-factor SPM-LS structures.

The main result of this study is that SPM-LS can be considered as essentially unidimensional measurement of intelligence if appropriately treated. Reliability and unidimensionality indices obtained from a bi-dimensional bi-factor model provided strong evidence of this conclusion. Notwithstanding the evidence of essential unidimensionality, it is also true that a non-ignorable, nuisance factor associated with the last six indicators of the SPM-LS was systematically found, either when applying parallel analysis, EGA, or factor modelling. An additional residual covariation between SPM4–SPM5 was also observed. This circumstance that should be discussed in more detail: Firstly, such a high residual correlation between both items might be due to significant estimation error in the tetrachoric matrix, altogether with the limited sample size. If so, future research employing different, larger samples should be able to identify a substantially smaller covariation between these items. Secondly, the relationship between SPM4 and SPM5 in terms of content and rules used for resolving these items should be inspected in further detail in order to decide if the information provided by both items is truly distinct or redundant.

This study evidence dimensionality assessment is a complex task which often requires convergent evidence from different sources and statistical techniques (as suggested in the case of parallel analysis and EGA; [17]). Moreover, being overconfident about model fit indices could be misleading when selecting an appropriate solution. Model fit should always be complemented with alternative indices (such as $\omega_{H}$, ECV or PUC) when possible [34]. Lastly, caution should be exercised when interpreting high inter-factor correlations in confirmatory models as evidence of unidimensionality, as these correlations could be inflated if relevant cross-loadings are being omitted. As an example, the inter-factor correlation was substantially larger for the bi-dimensional confirmatory structure that for its exploratory counterpart. To avoid such situations, we recommend researchers to confront results from both exploratory and confirmatory versions of the models to be investigated. If relevant cross-loadings to be potentially fixed are identified, we agree with previous authors that exploratory models should be prioritized [19,20].

Lastly, the result of applying bi-factor modelling was clear: We found evidence of a robust and reliable *g* factor (which resulted in our conclusion of SPM-LS scores being essentially unidimensional by current benchmarks [34]), plus an additional nuisance factor related with the last six items. While the interpretation of this latter factor could be somewhat controversial, it cannot be associated with a speed factor as in previous applications of similar tests [7,56] (as respondents had no time limit to reply to the matrices). An alternative explication is that such a factor would be related to guessing strategy or a difficulty component. Noteworthy, the first six items were (almost uniformly) correctly responded (with a proportion of correct responses near to 0.80), with the last six items presented a decreasing proportion of right answered (as evidenced in Figure 2). Under these conditions, it is known that parallel analysis is set to fail and that exploratory factor analysis under tetrachoric correlations could result in reflecting a difficulty factor [57,58]. Alternatively, the idea of guessing strategies being a relevant aspect of SPM-LS data was strongly supported by the original authors [2], as they showed that a three-parameter IRT model (incorporating a pseudo-guessing parameter) fitted the data better than alternative models. In this sense, and as pointed out by a reviewer, statistical artefacts of similar nature could be observed when applying factor analysis to a tetrachoric correlation matrix obtained from data generated from a three-parameter IRT model. Therefore, additional research on this matter should be granted in future SPM-LS applications. Thus, evidence suggests that guessing could play a substantive role with regards to general intelligence estimation. Even though we expanded these findings by identifying that guessing could also affect dimensionality assessment, future research should focus on re-assessing SPM-LS dimensionality under the assumption of data being generated from the three-parameter nested logistic model, as it has been shown to improve the effectiveness of parallel analysis [58]. Lastly, specific item position and item difficulty effects should aim to be separately studied (as they are confounded in the current SPM-LS form). Additionally, structural models aimed to measure each specific effect should also be encouraged to be applied [14].

Overall, the consequences of the presented findings are two-folded: firstly, even though researchers could treat SPM-LS as essentially unidimensional, this does not preclude them to not use the better measurement model (i.e., the bi-factor form) in their statistical analyses, especially if included within an SEM framework. Failing to take the influence of the second factor into account could lead to inflating or deflated regression coefficient and other types of measurement error propagation [39]. As an example, in our results, the variance explained by the second factor is of 0.17. If we assume a criterion Y, measured with reliability of one and a perfect positive relationship with the nuisance factor, the expected value for the estimated correlation between our nuisance factor and Y would be estimated as 0.41 (considering the attenuation by reliability described in [59]). Even though such distorting effect represents a worst-case scenario, where expected attenuation effects are anticipated to be smaller (as either criterion reliability or true relationship between criterion or specific factor would be not perfect), they should not be disregarded as negligible [59].

An attenuation of this magnitude could impact the evaluation of SPM-LS scores criterion and incremental validity (the expected increment of the determination coefficient might range from zero to 0.17). Note that our analysis identifies a source of performance variance. The effects might be even more substantial for a group with larger variance in the secondary factor. Consequently, despite the essential unidimensionality of the measure, the consequences of taking or not this second factor into account must be weighted in future research endeavours, including additional intelligence and ability measures.

Secondly, and from a theoretical point of view, researchers should not automatically disregard such secondary factors, as they could be tied to relevant individual differences of the test-takers [15,16]. On the contrary, more research is needed for us to have a better understating of the nature of this nuisance factor, and the extent that it could represent valuable information of the examinees.

## 5. Conclusions

The SPM-LS has been suggested to be a valid, reliable alternative version of the Standard Progressive Matrices test, presenting superior psychometric properties to alternatives such as the Advanced Progressive Matrices test. In this research, we provided a detailed study of the essential unidimensionality claimed by the original authors by utilising applying modern dimensionality techniques and bi-factor modelling. Our results suggest that, if appropriately treated, SPM-LS scores can be considered as such. Nevertheless, an additional factor relevant to the last six items was identified. Additionally, we recommend evaluating further the presence of this factor in additional, larger sample sizes presenting more balanced responses to the SPM-LS test.

## Figures and Tables

**Table 1:** Standardized factor loadings for all model tested.

	Unidim.	Unidim.M.	BID.EFA		BID.CFA		BEFA		BCFA	
Item	G	G	S1	S2	S1	S2	G	S1	G	S
SPM1	**0.47**	**0.48**	**0.59**	−0.08	**0.50**	0.00	**0.54**	−0.12	**0.50**	0.00
SPM2	**0.72**	**0.74**	**0.93**	−0.15	**0.76**	0.00	**0.82**	−0.22	**0.77**	0.00
SPM3	**0.72**	**0.73**	**0.88**	−0.09	**0.76**	0.00	**0.81**	−0.16	**0.76**	0.00
SPM4	**0.92**	**0.84**	**0.55**	**0.41**	**0.89**	0.00	**0.81**	0.25	**0.88**	0.00
SPM5	**0.94**	**0.87**	**0.67**	**0.30**	**0.91**	0.00	**0.86**	0.15	**0.91**	0.00
SPM6	**0.81**	**0.83**	**0.75**	0.17	**0.85**	0.00	**0.85**	0.05	**0.85**	0.00
SPM7	**0.66**	**0.67**	**0.30**	**0.47**	0.00	**0.71**	**0.60**	**0.33**	**0.62**	**0.30**
SPM8	**0.70**	**0.71**	0.23	**0.58**	0.00	**0.75**	**0.61**	**0.42**	**0.62**	**0.40**
SPM9	**0.61**	**0.61**	0.20	**0.50**	0.00	**0.65**	**0.53**	**0.36**	**0.52**	**0.39**
SPM10	**0.79**	**0.80**	**0.43**	**0.48**	0.00	**0.85**	**0.74**	**0.32**	**0.76**	0.27
SPM11	**0.62**	**0.63**	−0.04	**0.75**	0.00	**0.66**	**0.44**	**0.58**	**0.44**	**0.63**
SPM12	**0.53**	**0.54**	**−0.38**	**1.00**	0.00	**0.57**	0.28	**0.80**	**0.31**	**0.73**
φ	-	-	0.56		0.82		0.00		0.00	
SPM4-SPM5	-	0.69	0.70		0.56		0.70		0.57	

^1^ Unidim = Unidimensional model. Unidim.M. = Unidimensional model with SPM4-SPM5 residual correlation estimated. BID.EFA = Bi-dimensional exploratory factor analysis. BID.CFA = Bi-dimensional confirmatory factor analysis. BEFA = Bi-factor exploratory factor analysis. BCFA = Bi-factor confirmatory factor analysis. All loadings over 0.30 are presented bolded. φ = Inter-factor correlation. SPM4–SPM5 = Residual covariance between SPM4-SPM5 items. G = General Factor. S1= First specific factor. S2 = Second specific factor. Factor loadings with values > 0.30 appear bolded.

**Table 2:** Model fit indices for all tested models.

	Np	df	${Χ}^{2}$	*p*	CFI	TLI	RMSEA	SRMR
Unidim.	24	54	221.75	0.00	0.95	0.93	0.08 (0.07–0.09)	0.11
Unidim.M.	25	53	205.88	0.00	0.95	0.94	0.08 (0.07–0.08)	0.11
BID.EFA/BEFA.	36	42	**80.50**	**0.00**	**0.99**	**0.98**	**0.04 (0.03–0.06)**	**0.06**
BID.CFA	26	52	160.69	0.00	0.96	0.96	0.07 (0.05–0.08)	0.09
BCFA	31	47	113.72	0.00	0.98	0.97	0.05 (0.04, 0.07)	0.07

^1^ Unidim = Unidimensional model. Unidim.M. = Unidimensional model with SPM4–SPM5 residual correlation estimated. BID.EFA = Bi-dimensional exploratory factor analysis. BID.CFA = Bi-dimensional confirmatory factor analysis. BEFA = Bi-factor exploratory factor analysis. BCFA = Bi-factor confirmatory factor analysis. Np = Estimated number of parameters. Df = degrees of freedom. ${Χ}^{2}\;$ = Chi-square statistic. P = *p*-value associated with ${Χ}^{2}$ test of fit. CFI = Comparative fit index. TLI = Tucker-Lewis index. RMSEA = Root Mean Square Error of Approximation (with 95% confidence interval in parenthesis). SRMS = Standardized Root Mean Square Residual. Best fit indices presented bolded and underlined. Model fit indices for the best fitting model appear bolded.

**Figure A1:**
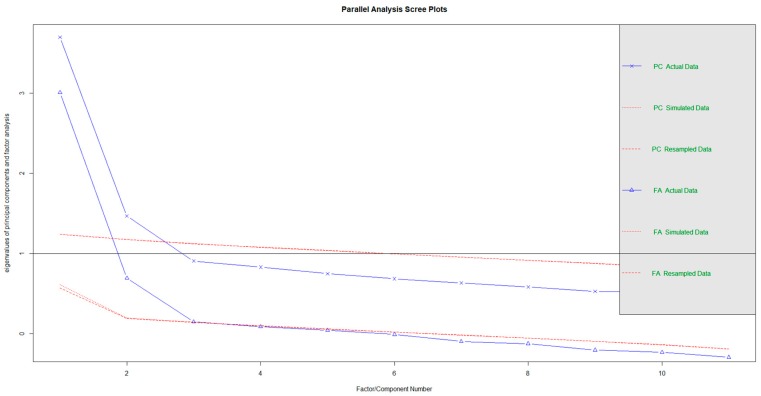
Principal component and parallel factor analysis with eigenvalue obtained from resampling from original data using a parcel for SPM4 and SPM5 items.

**Figure A2:**
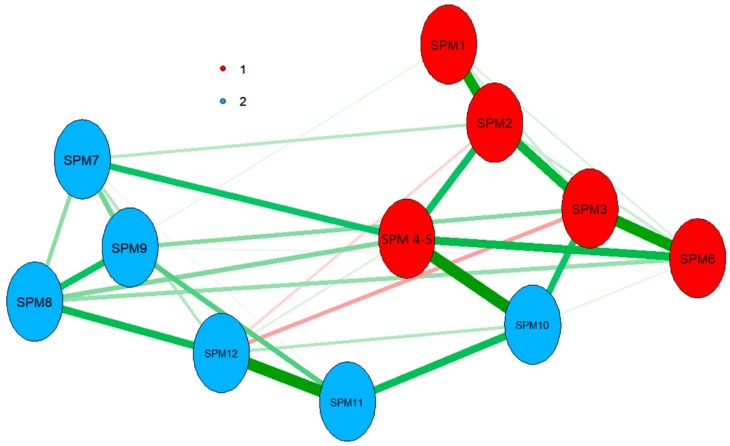
Exploratory Graph Analysis of SPM-LS data with SPM4 and SPM5 item combined. Dimensions and items associated are presented in different colours. Positive partial correlations are depicted in green, with negative partial correlations presented in red. The size of the lines indicates the size of the partial correlations. SPM4 = SPM4-5 item.

## Footnotes

1 Specific factor omega hierarchical and PUC are only computable for confirmatory solutions. Estimating such statistics in exploratory models would require researchers to decide which items or correlations are being considered by the specific factors.

2 Using other extraction methods (i.e., ordinary least squares) led to similar conclusions regarding the underlying dimensionality, but for weighted and generalized least squares, which suggested to retain three factors and two components.

3 Using alternative oblique rotations (i.e., oblimin, promax, geomin) resulted in factor structures with a similar distribution of loadings and size. Main differences were small in magnitude, and mostly affected the inter-factor correlation size.

## Supplementary Materials

The following are available online at https://www.mdpi.com/2079-3200/7/3/14/s1.

## Appendix A

In this Appendix A, the SPM-LS dimensionality will be re-analysed by including a parcel created by aggerating SPM4-SPM5 items. This decision was taken based on the high dependence observed between items SPM4–SPM5 (i.e., tetrachoric correlation of 0.91; high partial correlation detected in EGA) Thus, we will follow the same steps performed in the primary analysis. Firstly, we reproduce the tetrachoric-polychoric correlation analysed in these analyses. As expected, most correlations between items and the combined item (i.e., SPM4-5) were like the original (Table A1).

**Table A1:** Tetrachoric/polychoric correlation matrix with SPM4 and SPM5 combined.

	1	2	3	4–5	6	7	8	9	10	11	12
SPM1	1										
SPM2	0.59	1									
SPM3	0.47	0.69	1								
SPM4-5	0.40	0.67	0.54	1							
SPM6	0.44	0.62	0.73	0.72	1						
SPM7	0.23	0.48	0.38	0.62	0.48	1					
SPM8	0.32	0.40	0.41	0.60	0.51	0.53	1				
SPM9	0.13	0.36	0.48	0.41	0.47	0.49	0.55	1			
SPM10	0.28	0.46	0.63	0.77	0.61	0.48	0.49	0.46	1		
SPM11	0.25	0.25	0.31	0.42	0.42	0.42	0.44	0.49	0.59	1	
SPM12	0.13	0.06	0.04	0.43	0.29	0.41	0.52	0.37	0.45	0.61	1

We performed principal components, and factor analysis parallel analysis with eigenvalues resampled from the original data over this correlation matrices. Both techniques agreed to indicate that the structure was bi-dimensional (Figure A2). The value of the original components was 3.70 and 1.47 (with resampled components of 1.24 and 1.17), and the value of the original factor was 3.01 and 0.69 (with resampled eigenvalues of 0.64 and 0.19).

EGA agreed with parallel analysis results and concluded that two dimensions are underlying the SPM-LS scores if SPM4 and SPM5 items were combined. Thus, there was robust evidence of the bi-dimensional nature of the data after controlling for the dependency between SPM4 and SPM5 items.

Lastly, and in the case to be of interest, standardised factor loadings and model fit indices are provided. Noteworthy, results were similar to other models presented in this article but provided a sustainably worse fit to the data. In the exploratory models, SPM4-5 showed lower factor loadings in the S1 (model BID.EFA) or G (model BEFA), and higher cross-loadings on the alternative factors. In the confirmatory models, SPM4-5 loadings were also closer to 0.90 than in the main text results. Overall, resulting structures were mostly similar to those analysed in the result section of the article.

**Table A2:** Standardised factor loadings for all model tested.

	Unidim.	BID.EFA		BID.CFA		BEFA		BCFA	
Item	G	S1	S2	S1	S2	G	S1	G	S
SPM1	**0.47**	**0.58**	−0.04	**0.50**	0.00	**0.55**	−0.10	**0.51**	0.00
SPM2	**0.72**	**0.90**	−0.10	**0.76**	0.00	**0.84**	−0.17	**0.77**	0.00
SPM3	**0.74**	**0.87**	−0.04	**0.77**	0.00	**0.84**	−0.13	**0.79**	0.00
SPM4-5	**0.85**	**0.55**	**0.43**	**0.90**	0.00	**0.82**	0.28	**0.90**	0.00
SPM6	**0.82**	**0.70**	0.22	**0.86**	0.00	**0.84**	0.10	**0.85**	0.00
SPM7	**0.67**	**0.26**	**0.51**	0.00	**0.70**	**0.57**	**0.36**	**0.60**	**0.31**
SPM8	**0.71**	0.20	**0.60**	0.00	**0.74**	**0.58**	**0.45**	**0.61**	**0.41**
SPM9	**0.62**	0.19	**0.52**	0.00	**0.65**	**0.52**	**0.38**	**0.52**	**0.40**
SPM10	**0.80**	**0.40**	**0.51**	0.00	**0.84**	**0.72**	**0.35**	**0.75**	0.29
SPM11	**0.64**	−0.04	**0.75**	0.00	**0.67**	**0.43**	**0.59**	**0.45**	**0.61**
SPM12	**0.54**	**−0.39**	**1.00**	0.00	**0.57**	0.23	**0.82**	0.29	**0.76**
φ	**-**	**0.54**		**0.81**		0.00		0.00	

^1^ Unidim = Unidimensional model. Unidim.M. = Unidimensional model with SPM4-SPM5 residual correlation estimated. BID.EFA = Bi-dimensional exploratory factor analysis. BID.CFA = Bi-dimensional confirmatory factor analysis. BEFA = Bi-factor exploratory factor analysis. BCFA = Bi-factor confirmatory factor analysis. All loadings over 0.30 are presented bolded. φ = Inter-factor correlation. SPM4-SPM5 = Residual covariance between SPM4-SPM5 items. G = General Factor. S1= First specific factor. S2 = Second specific factor. Factor loadings with values > 0.30 appear bolded.

**Table A3:** Model fit indices for all tested models.

	Np	df	${Χ}^{2}$	*p*	CFI	TLI	RMSEA	SRMR
Unidim.	23	44	192.04	0.00	0.93	0.91	0.08 (0.07–0.09)	0.11
BID.EFA/BEFA.	33	34	68.45	0.00	**0.98**	**0.97**	**0.05 (0.03–0.06)**	**0.05**
BID.CFA	24	43	145.71	0.00	0.95	0.94	0.07 (0.06–0.08)	0.09
BCFA	29	38	110.79	0.00	0.97	0.96	0.06 (0.04–0.07)	0.07

^1^ Unidim = Unidimensional model. Unidim.M. = Unidimensional model with SPM4-SPM5 residual correlation estimated. BID.EFA = Bi-dimensional exploratory factor analysis. BID.CFA = Bi-dimensional confirmatory factor analysis. BEFA = Bi-factor exploratory factor analysis. BCFA = Bi-factor confirmatory factor analysis. Np = Estimated number of parameters. Df = degrees of freedom. ${Χ}^{2}$ = Chi-square statistic. P = *p*-value associated with ${Χ}^{2}$ test of fit. CFI = Comparative fit index. TLI= Tucker-Lewis index. RMSEA = Root Mean Square Error of Approximation (with 95% confidence interval in parenthesis). SRMS = Standardized Root Mean Square Residual. Best fit indices presented bolded and underlined. Model fit indices for the best fitting model appear bolded.

## Appendix B

In Appendix B, standardised factor loadings (Table A4) and model fit indices (Table A5) are provided for models without the residual correlation SPM4-SPM5.

**Table A4:** Standardised factor loadings for all model tested.

	Unidim.	BID.EFA		BID.CFA		BEFA		BCFA	
Item	G	S1	S2	S1	S2	G	S1	G	S
SPM1	**0.47**	**0.59**	−0.09	**0.50**	0.00	**0.53**	−0.13	**0.50**	0.00
SPM2	**0.72**	**0.93**	−0.18	**0.75**	0.00	**0.81**	−0.23	**0.76**	0.00
SPM3	**0.72**	**0.87**	−0.10	**0.75**	0.00	**0.80**	−0.16	**0.76**	0.00
SPM4	**0.92**	**0.65**	**0.38**	**0.94**	0.00	**0.90**	0.22	**0.93**	0.00
SPM5	**0.94**	**0.74**	**0.30**	**0.95**	0.00	**0.94**	0.16	**0.96**	0.00
SPM6	**0.81**	**0.73**	0.15	**0.84**	0.00	**0.83**	0.04	**0.84**	0.00
SPM7	**0.66**	**0.30**	**0.47**	0.00	**0.71**	**0.59**	**0.33**	**0.61**	**0.31**
SPM8	**0.70**	0.23	**0.57**	0.00	**0.75**	**0.60**	**0.42**	**0.61**	**0.41**
SPM9	**0.60**	0.20	**0.50**	0.00	**0.64**	**0.52**	**0.36**	**0.51**	**0.40**
SPM10	**0.79**	**0.43**	**0.47**	0.00	**0.84**	**0.73**	**0.31**	**0.75**	**0.30**
SPM11	**0.62**	-0.05	**0.75**	0.00	**0.66**	**0.44**	**0.58**	**0.44**	**0.64**
SPM12	**0.53**	**−0.36**	**0.99**	0.00	**0.57**	0.28	**0.80**	**0.31**	**0.72**
φ	**-**	**0.57**		**0.80**		0.00		0.00	

^1^ Unidim = Unidimensional model. Unidim.M. = Unidimensional model with SPM4-SPM5 residual correlation estimated. BID.EFA = Bi-dimensional exploratory factor analysis. BID.CFA = Bi-dimensional confirmatory factor analysis. BEFA = Bi-factor exploratory factor analysis. BCFA = Bi-factor confirmatory factor analysis. All loadings over 0.30 are presented bolded. Phi = Inter-factor correlation. SPM4-SPM5 = Residual covariance between SPM4-SPM5 items. G = General Factor. S1= First specific factor. S2 = Second specific factor. Factor loadings with values > 0.30 appear bolded.

**Table A5:** Model fit indices for all tested models.

	Np	df	${Χ}^{2}$	*p*	CFI	TLI	RMSEA	SRMR
Unidim.	24	54	221.75	0.00	0.94	0.93	0.08 (0.08–0.09)	0.11
BID.EFA/BEFA.	35	43	97.21	0.00	**0.98**	**0.97**	**0.05 (0.04–0.06)**	**0.06**
BID.CFA	25	53	163.39	0.00	0.96	0.96	0.07 (0.05–0.07)	0.09
BCFA	30	48	117.65	0.00	0.98	0.97	0.05 (0.04–0.07)	0.07

^1^ Unidim = Unidimensional model. Unidim.M. = Unidimensional model with SPM4-SPM5 residual correlation estimated. BID.EFA = Bi-dimensional exploratory factor analysis. BID.CFA = Bi-dimensional confirmatory factor analysis. BEFA = Bi-factor exploratory factor analysis. BCFA = Bi-factor confirmatory factor analysis. Np = Estimated number of parameters. Df = degrees of freedom. ${Χ}^{2}$ = Chi-square statistic. P = *p*-value associated with ${Χ}^{2}$ test of fit. CFI = Comparative fit index. TLI= Tucker-Lewis index. RMSEA = Root Mean Square Error of Approximation (with 95% confidence interval in parenthesis). SRMS = Standardized Root Mean Square Residual. Best fit indices presented bolded and underlined. Model fit indices for the best fitting model appear bolded.

## References

[1] Raven J.C. (1941). Standardization of Progressive Matrices. Br. J. Med. Psychol..

[2] Myszkowski N., Storme M. (2018). A snapshot of g? Binary and polytomous item-response theory investigations of the last series of the Standard Progressive Matrices (SPM-LS). Intelligence.

[3] Abad F.J., Colom R., Rebollo I., Escorial S. (2004). Sex differential item functioning in the Raven’s Advanced Progressive Matrices: Evidence for bias. Pers. Indiv. Differ..

[4] Lucio P.S., Cogo-Moreira H., Puglisi M., Polanczyk G.V., Little T.D. (2017). Psychometric Investigation of the Raven’s Colored Progressive Matrices Test in a Sample of Preschool Children. Assessment.

[5] Walsch N.A., Nettelbeck S.A.J., Nicholas R.B. (2016). Dimensionality of the Raven’s Advanced Progressive Matrices: Sex Differences and Visuospatial Ability. Pers. Individ. Differ..

[6] Lohmann D.F., Lakin J.M., Sternberg R.J., Kaufman S.B. (2011). Intelligence and Reasoning. The Cambridge Handbook of Intelligence.

[7] Dillon R.F., Pohlmann J.T., Lohman D. (1981). A Factor Analysis of Raven’s Advanced Progressive Matrices Freed from Difficulty Factors. Educ. Psychol. Meas..

[8] Bors D.A., Stokes T.L. (1998). Raven’s Advanced Progressive Matrices: Norms for First-Year University Students and the Development of a Short Form. Educ. Psychol. Meas..

[9] Holzinger K., Swineford F. (1937). The Bi-factor Method. Psychometrika.

[10] Waller N.G. (2017). Direct Schmid-Leiman Transformations and Rank-Deficient Loadings Matrices. Psychometrika.

[11] Johnson W., Bouchard T.J. (2011). The MISTRA data: Forty-two mental ability tests in three batteries. Intelligence.

[12] Gignac G.E. (2015). Raven’s is not a pure measure of general intelligence: Implications for g factor theory and the brief measurement of g. Intelligence.

[13] Muniz M., Gomez C., Pasian S. (2016). Factor Structure of Raven’s Coloured Progressive Matrices. Psico-USF.

[14] Zeller F., Reiß S., Schweizer K. (2019). Is the Item-Position Effect in Achievement Measures Induced by Increasing Item Difficulty. Struct. Equ. Model..

[15] Ren X., Wang T., Sun S., Deng M., Scheizer K. (2018). Speeded testing in the assessment of intelligence gives rise to speed factor. Intelligence.

[16] Sun S., Scheizer K., Ren X. (2019). Item-Position Effect in Raven’s Matrices: A Developmental Perspective. J. Cogn. Dev..

[17] Golino H.F., Shi D., Garrido L.E., Christensen A., Nieto M.D., Sadana P., Thiyagarajan J.A. (2019). Investigating the performance of exploratory graph analysis and traditional techniques to identify the number of latent factors: A simulation and tutorial. Psychol. Methods.

[18] Lubbe D. (2018). Parallel Analysis with Categorical Variables: Impact of Category Probability Proportions on Dimensionality Assessment Accuracy. Psychol. Methods.

[19] Marsh H., Morin A., Parker P., Kaur G. (2014). Exploratory structural equation modeling: An integration of the best features of exploratory and confirmatory factor analysis. Annu. Rev. Clin. Psychol..

[20] Marsh H., Muthen B., Asparouhov T., Lüdke O., Robitzsch A., Morin A., Trautwein U. (2009). Exploratory structural equation modelling, integrating CFA and EFA: Application to student’s evaluations of university teaching. Struct. Equ. Model..

[21] Revelle W., Wilt J. (2013). The general factor of personality: A general critique. J. Res. Pers..

[22] Timmerman M.E., Lorenzo-Seva U. (2016). Dimensionality Assessment of Ordered Polytomous Items with Parallel Analysis. Psychol. Methods.

[23] Garrido L.E., Abad F.J., Ponsoda V. (2016). Are fit indices really fit to estimate the number of factors with categorical variables? Some cautionary findings via Monte Carlo simulation. Psychol. Methods.

[24] Garrido L.E., Abad F.J., Ponsoda V. (2013). A new look at Horn’s parallel analysis with ordinal variables. Psychol. Methods.

[25] Raiche G., Walls T., Magis D., Riopel M., Blais J.G. (2013). Non-graphical Solutions for Cattell’s Scree Test. Methodology.

[26] Crawford A.V., Green S.B., Levy R., Lo W.J., Scott L., Svetina D., Thompson M.S. (2010). Evaluation of parallel analysis methods for determining the number of factors. Educ. Psychol. Meas..

[27] Parry D.H., McArdle J.J. (1991). An Applied Comparison of Methods for Least-Squares Factor Analysis of Dichotomous Variables. Appl. Psychol. Meas..

[28] Weng L.J., Cheng C.P. (2005). Parallel Analysis with Unidimensional Binary Data. Educ. Psychol. Meas..

[29] Mulaik S. (2010). Foundations of Factor Analysis.

[30] Golino H.F., Epskamp S. (2017). Exploratory Graph Analysis: A New Approach for Estimating the Number of Dimensions in Psychological Research. PLoS ONE.

[31] Golino H.F., Demetriou A. (2017). Estimating the Dimensionality of Intelligence like Data using Exploratory Graph Analysis. Intelligence.

[32] Rodriguez A., Reise S.P., Haviland M.G. (2016). Applying Bifactor Statistical Indices in the Evaluation of Psychological Measures. J. Pers. Assess..

[33] Rodriguez A., Reise S.P., Haviland M.G. (2016). Evaluating bifactor models: Calculating and interpreting statistical indices. Psychol. Methods.

[34] Reise S.P., Scheines R., Widaman K., Haviland M. (2013). Multidimensionality and Structural Coefficient Bias in Structural Equation Modelling: A Bifactor Perspective. Educ. Psychol. Meas..

[35] Reise S.P., Bonifay W., Haviland M.G., Irwing P., Booth T., Hughes D.J. (2018). Bifactor modelling and the evaluation of scale scores. The Wiley Handbook of Psychometric Testing: A Multidisciplinary Reference on Survey, Scale and Test Development.

[36] Garcia-Garzon E., Abad F.J., Garrido L.E. (2019). Improving Bi-factor Exploratory Modelling: Empirical Target Rotation Based on Loading Differences. Methodology.

[37] Mai Y., Zhang Z., Wen Z. (2018). Comparing Exploratory Structural Equation Modeling and Existing Approaches for Multiple Regression with Latent Variables. Struct. Equ. Model..

[38] Abad F.J., Garcia-Garzon E., Garrido L.E., Barrada J.R. (2017). Iteration of Partially Specified Target Matrices: Application to the Bi-Factor Case. Multivar. Behav. Res..

[39] Asparouhov T., Muthen B. (2009). Exploratory Structural Equation Modeling. Struct. Equ. Model..

[40] Mansolf M., Reise S.P. (2016). Exploratory Bifactor Analysis: The Schmid-Leiman Orthogonalization and Jennrich-Bentler Analytic Rotations. Multivar. Behav. Res..

[41] Lorenzo-Seva U., Ferrando P.J. (2019). A General Approach for Fitting Pure Exploratory Bifactor Models. Multivar. Behav. Res..

[42] Lorenzo-Seva U. (1999). Promin: A Method for Oblique Factor Rotation. Multivar. Behav. Res..

[43] Jennrich R.I., Bentler P. (2011). Exploratory Bi-factor Analysis. Psychometrika.

[44] Jennrich R.I., Bentler P. (2012). Exploratory Bi-factor Analysis: The Oblique Case. Psychometrika.

[45] R Core Team (2019). R: A Language and Environment for Statistical Computing.

[46] Allaire J.J., Xie Y., McPherson J., Luraschi J., Ushey K., Atkins A., Wickham H., Cheng J., Chang, Iannone R. (2019). Rmarkdown: Dynamic Documents for R. https://rmarkdown.rstudio.com.

[47] Aust F., Barth M. (2018). Papaja: Prepare Reproducible APA Journal Articles with R Markdown. https://github.com/crsh/papaja.

[48] Epskamp S., Cramer A.O.J., Waldorp L.J., Schmittmann V.D., Borsboom D. (2012). Qgraph: Network Visualizations of Relationships in Psychometric Data. J. Stat. Softw..

[49] Revelle W. (2018). Psych: Procedures for Personality and Psychological Research.

[50] Golino H. (2019). EGA: Exploratory Graph Analysis: Estimating the Number of Dimensions in Psychological Data. http://github.com/hfgolino/EGA.

[51] Rosseel Y. (2012). lavaan: An R Package for Structural Equation Modeling. J. Stat. Softw..

[52] Jorgensen T.D., Pornprasertmanit S., Schoemann A.M., Rosseel Y. (2018). SemTools: Useful Tools for Structural Equation Modeling. https://CRAN.R-project.org/package=semTools.

[53] Viladrich C., Angulo-Brunet A., Doval E. (2017). A Journey Around Alpha and Omega to Estimate Internal Consistency Reliability. Ann. Psychol..

[54] Bernaards C.A., Jennrich R.I. (2005). Gradient Projection Algorithms and Software for Arbitrary Rotation Criteria in Factor Analysis. Educ. Psychol. Meas..

[55] Little T.D., Rhemtulla M., Gibson K., Schoemann A.M. (2013). Why the Items versus Parcels Controversy Needn’t Be one. Psychol. Methods.

[56] Estrada E., Román F.J., Abad F.J., Colom R. (2017). Separating power and speed components of standardized intelligence measures. Intelligence.

[57] Carroll J.B. (1945). The effect of difficulty and chance success on correlations between items or between tests. Psychometrika.

[58] DeMars C.E. (2019). Revised Parallel Analysis with Nonnormal Ability and a Guessing Parameter. Educ. Psychol. Meas..

[59] Abad F.J., Sorrel M.A., Garcia L.F., Aluja A. (2018). Modeling General, Specific, and Method Variance in Personality Measures: Results for ZKA-PQ and NEO-PI-R. Assessment.

